# Long-term survival of a woman with well differentiated papillary mesothelioma of the peritoneum: a case report and review of the literature

**DOI:** 10.1186/1752-1947-4-346

**Published:** 2010-10-29

**Authors:** Jeffrey M Clarke, Paul Helft

**Affiliations:** 1 Department of Medicine, Duke University Medical Center, Durham, NC, USA; 2 Department of Medicine, Section of Hematology/Oncology, Indiana University Melvin and Bren Simon Cancer Center, Indiana University School of Medicine, Indianapolis, IN, USA

## Abstract

**Introduction:**

Well-differentiated papillary mesothelioma of the peritoneum (WDPMP) is a rare subtype of epitheloid mesothelioma, which is usually seen in young women. WDPMP is generally considered of low malignant potential, however the long-term nature of the tumor remains poorly defined.

**Case presentation:**

We describe the long-term follow-up of a 60-year-old woman of West African descent who has survived 24 years with WDPMP after receiving extensive local and systemic adjuvant chemotherapy. Her clinical course has included three exploratory laparotomies with intraperitoneal and intravenous chemotherapy over two decades. Her course was complicated by anthracycline-induced cardiomyopathy, for which she underwent an orthotopic heart transplant. Our patient is alive with stable radiological evidence of peritoneal disease, and continues to suffer from chronic abdominal pain.

**Conclusion:**

No consensus exists regarding optimal treatment strategies for WDPMP. However, given the low malignant potential of the tumor, careful consideration should be made before proceeding with aggressive interventions. Further, long-term follow-up reports are required to fully characterize this tumor.

## Introduction

Mesothelioma is an uncommon neoplasm which originates from the mesothelial lining of the pleura, pericardium, peritoneum, and tunica vaginalis [1,2]. Malignant peritoneal mesothelioma (MPM) makes up approximately 10% to 20% of all cases of mesothelioma [2]. MPM is an aggressive tumor typically associated with asbestos exposure and afflicts mainly men in the fifth to sixth decades of life [2,3]. In contrast, well-differentiated papillary mesothelioma of the peritoneum (WDPMP) is a rare subtype of epitheloid mesothelioma, which is usually seen in young women [1,4,5]. WDPMP is generally considered of low malignant potential and falls within a clinicohistological spectrum of papillary peritoneal tumors in women ranging from mesothelial hyperplasia to papillary carcinoma [1,5]. While the histological features of WDPMP have been described in many cases with short-term clinical follow-up, the long-term nature of the tumor remains poorly defined. We present a case describing long-term survival and follow-up of woman with WDPMP who received extensive intraperitoneal and systemic chemotherapy.

## Case presentation

We report the case of a 60-year-old woman of West African descent, with no history of asbestos exposure, who originally presented 24 years ago to another institution with acute abdominal pain. At that time, she underwent an exploratory laparotomy and was found to have nodules diffusely covering the peritoneum. A total abdominal hysterectomy and bilateral salpingo-oophorectomy were performed for suspected ovarian carcinoma, and biopsies were taken of the peritoneal nodules. The pathology from this original surgery was interpreted as low-grade papillary mesothelioma. She then received six adjuvant cycles of intravenous cyclophosphamide, doxorubicin and cisplatin. She underwent a second-look laparotomy six months later, and still had gross disease visible in the peritoneum. Post-operatively she received three additional cycles of intraperitoneal cisplatin and intravenous sodium thiosulfate. She subsequently received maintenance therapy with alternating courses of tamoxifen and megace alternating every two weeks.

She presented four years later with obstructive gastrointestinal symptoms and was again found on laparotomy to have diffuse peritoneal studding. Pathology from this surgery was interpreted again to be papillary mesothelioma. As a result, she began six cycles of carboplatin and cyclophosphamide chemotherapy for suspected progressive disease. Several months later, she presented with complaints of shortness of breath, orthopnea, and worsening lower extremity edema. A multi-gated acquisition scan (MUGA) revealed an ejection fraction of 14% and enlarged cardiac silhouette on chest X-ray, and she was clinically diagnosed as having anthracycline-induced cardiomyopathy. Medical therapy was initiated at that time for congestive heart failure.

Two years later, she was found on a computed topography (CT) scan to have an interval increase in loculated subhepatic fluid collection and a lobular soft tissue mass in the right subphrenic region. She then received three cycles of VP-16 and ifosfamide. She remained well until 2000, when she underwent an orthotopic heart transplant. Upon subsequent reimaging of her abdomen the next year, she was found to have continued slow progression of the tumor and was started on single-agent paclitaxel followed by cyclophosphamide for two months. She was then referred to our institution in late 2001 with stable disease on abdominal CT and a presumed diagnosis of malignant peritoneal mesothelioma refractory to therapy. Over the following year, she was maintained on combination capecitabine and gemcitabine therapy and had stable disease as assessed by CT scans. However, in early 2003 she was found to have declining renal function and was forced to stop chemotherapy.

**Figure 1:**
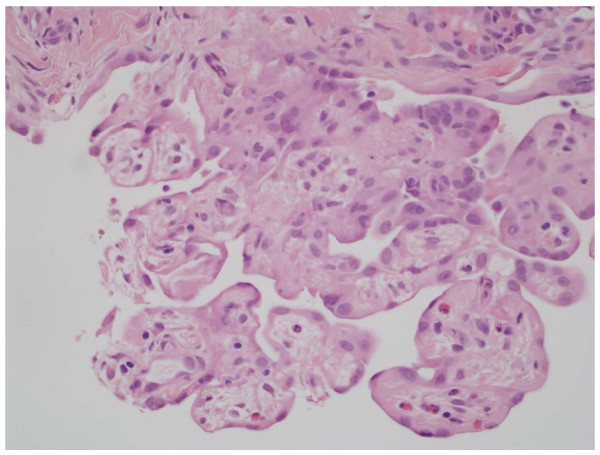
**Well differentiated papillary mesothelioma of the peritoneum (40× magnification)**. Multiple coarse papillae are present with varying fibrovascular cores and minimal cellular atypia.

**Figure 2:**
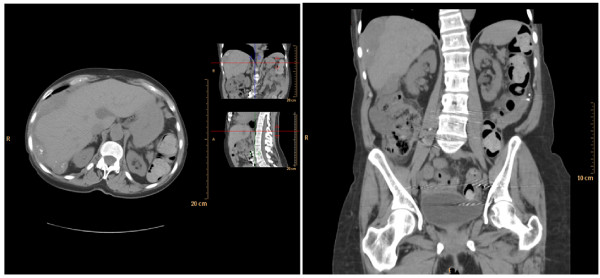
**Cross-sectional computed tomography (CT) images displaying a right-sided, perihepatic soft tissue mass containing calcifications and subhepatic loculated fluid collection**.

She was observed closely until 2004 and had little change in her overall tumor burden, but had recurrent ascites requiring drainage by paracentesis on multiple occasions. Because of doubts about the true nature of her peritoneal tumor, a further biopsy of her tumor was performed in 2004, with the final interpretation demonstrating a low-grade papillary mesothelioma of the peritoneum (see Figure 1). She has been observed closely since that time with periodic abdominal imaging showing a right side subphrenic mass, loculated subhepatic fluid collection, scattered soft tissue densities with calcification, and extensive anterior wall and peritoneal adhesive disease without obstruction (see Figure 2). She continues to have chronic renal insufficiency and suffers from severe chronic abdominal pain and cramping, but has stable radiological evidence of disease.

## Discussion

To the best of our knowledge, fewer than 60 cases of WDPMP have been described in the literature. The reported age at diagnosis has ranged from two to 74 years old [1,4]. Of 39 case reports we reviewed, the mean age at presentation was 44 years (median 43 years). In all, 28 patients were women and 11 patients were men [1,2,4,6-12]. Symptoms at presentation included acute and chronic abdominal pain, ascites, pleural effusion, bloating, weight loss, dyspareunia, and menorrhagia [1,4]. However, the diagnosis of WDPMP was frequently made incidentally during surgery [4]. Only six of the patients were reported to have possible asbestos exposure, but no definitive causation has ever been described [8,10]. Follow-up time was recorded for 37 of the 45 patient case reports we reviewed and ranged from six weeks to 29 years (median 36 months, mean 51 months) [1,2,4,6-12].

The reported cases of WDPMP retain several uniting histomorphological features. Coarse papillary architecture with fibrovascular cores is the most commonly seen appearance, with occasional areas of tubulopapillary pattern [1,4,7-10,12]. The papillae are lined by a simple uniform cuboidal epithelium, with little to no nuclear atypia or mitoses. The amount of fibrosis present can be variable and psammoma bodies can also be found [4,8]. Areas of invasion are typically not seen [4,5,8]. Microscopic analysis of cytology from ascites can show spheroid tumor cell clusters [12]. Classically, WDPMP exists within a spectrum of primary papillary peritoneal tumors described in women, which ranges from mesothelial hyperplasia to the more aggressive atypical diffuse mesothelioma and papillary carcinoma [1,5]. As suggested in several previous reports, the tumor must be distinguished from its benign and malignant counterparts based on degree of cellular differentiation and atypia [1,4,5,8,12].

The case presented above is unusual in two respects. She maintained follow-up and remains alive with disease after 24 years from her initial diagnosis. This is the second longest time of follow-up reported for WDPMP. The longest follow-up was a 41-year-old woman observed for 29 years who eventually died of a pancreatic carcinoma [4]. While many reported cases portray WDPMP as a clinically benign tumor, several case reports have described more aggressive behavior with long-term follow-up. In one case, a patient died five years following diagnosis. He was found at autopsy to have extensive retroperitoneal, anterior abdominal wall, diaphragmatic, and pericardial invasion, culminating in a large embolism of tumor cells to the pulmonary artery [7]. A second case describes a patient who died of diffuse malignant mesothelioma approximately nine years after the diagnosis of WDPMP, suggesting a malignant transformation at some point in the clinical course [9]. To better understand and characterize the malignant potential of this tumor, additional case reports with long-term follow-up are required.

Secondly, she received extensive chemotherapy with substantial associated morbidity, we believe on the basis of the fact that her tumor was thought originally to be an ovarian-derived tumor or primary peritoneal carcinoma, and was later thought to be a malignant peritoneal mesothelioma. Only after a repeat biopsy 19 years after her original diagnosis was the probable identity of her tumor finally understood.

Management of patients with WDPMP remains controversial. The majority of patients undergo initial exploratory laparotomy for diagnostic and cytoreductive purposes [1,4,8]. However this approach is contentious, given the low malignant potential of the tumor. Some authors recommend close observation or serial biopsy for surveillance [1]. Adjuvant treatment for WDPMP also remains poorly defined and was described in only 14 of the cases that we reviewed [2,4,8,10-12]. In the largest series, three patients received a combination of chemotherapy and radiation therapy, one of these with intravenous thiotepa, and two additional patients received radiation therapy alone [4]. Of the patients who received adjuvant radiation therapy, two patients had died of radiation enteritis and intestinal obstruction at two-year and seven-year follow-up, respectively [4]. Intraperitoneal administration of chemotherapy has been described in several case reports. One patient with simultaneous involvement of the pleural and peritoneal surfaces with ascites and pleural effusion was treated successfully with intraperitoneal, intrapleural, and intravenous carboplatin [12]. Our patient remained disease free at four years following presentation. Four patients have received intraperitoneal hyperthermic chemoperfusion (IPHP) therapy [2,6,10]. Two of these patients received cisplatin and doxorubicin following optimal debulking. One patient was alive with disease at 15 months, while the other patient suffered a post-operative colobronchial fistula requiring partial colectomy and was alive 40 months later [2,6]. Another patient received IPHP with cisplatin and mitomycin C after suboptimal debulking and died of disease progression 13 months later [2]. A third patient with concurrent rectal carcinoma underwent a low anterior resection with omentectomy and peritonectomy and subsequent IPHP with mitomycin-C and 5-fluorouracil [10]. Our patient had no evidence of disease at six months follow-up.

Five patients whose care reports we reviewed received intravenous chemotherapy alone, two of these with unspecified regimens [4,8,11]. Two patients received cisplatin and doxorubicin. One of these patients had no evidence of disease at three years, and the second patient died of disease three years later [8]. One case described an 11-year-old girl who was treated with combination cisplatin, cyclophosphamide and maintenance lupron [11]. She had stable diffuse peritoneal nodules at nine months. Considerable variability exists in the literature regarding the chemotherapeutic management of this tumor.

## Conclusion

Clearly, no consensus has been reached regarding optimal treatment strategies for WDPMP. It is difficult to determine the effect of systemic or intraperitoneal chemotherapy on the tumor progression of our patient due to imprecise past medical records. However, one must question the necessity of extensive chemotherapeutic and surgical therapies for a tumor with presumed low malignant potential, given the inherent risks of such interventions. Furthermore, accurate pathological diagnosis must be initially obtained in order to prevent overtreatment of WDPMP. Additional information obtained from other case reports describing the long-term behavior of this tumor should also help to elucidate the precise roles for observation and therapeutic intervention.

## Abbreviations

(MPM): Malignant peritoneal mesothelioma; (WDPMP): Well-differentiated papillary mesothelioma of the peritoneum; (MUGA): Multi-gated acquisition scan; (CT): Computed topography; (IPHP): Intraperitoneal hyperthermic chemoperfusion;

## Competing interests

The authors declare that they have no competing interests.

## Consent

Written informed consent was obtained from the patient for publication of this case report and any accompanying images. A copy of the written consent is available for review by the journal's Editor-in-Chief.

## Authors' contributions

JC researched and composed the literature review and the patient history. PH was a major contributor to the patient history and to critical revision of the manuscript. Both authors read and approved the final manuscript.
